# MiR-10a-5p targets TFAP2C to promote gemcitabine resistance in pancreatic ductal adenocarcinoma

**DOI:** 10.1186/s13046-018-0739-x

**Published:** 2018-04-03

**Authors:** Guangbing Xiong, Hua Huang, Mengyu Feng, Gang Yang, Suli Zheng, Lei You, Lianfang Zheng, Ya Hu, Taiping Zhang, Yupei Zhao

**Affiliations:** 1 Department of General Surgery, Peking Union Medical College Hospital, Chinese Academy of Medical Sciences and Peking Union Medical College, No. 1 Shuaifuyuan, Wangfujing Street, Beijing, 100730 China; 2 Department of Biliary-Pancreatic Surgery, Affiliated Tongji Hospital, Tongji Medical College, Huazhong University of Science and Technology, Wuhan, 430030 Hubei Province China; 3 Department of Nuclear Medicine, Peking Union Medical College Hospital, Chinese Academy of Medical Sciences and Peking Union Medical College, Beijing, 100730 China; 4 Clinical Immunology Center, Chinese Academy of Medical Sciences and Peking Union Medical College, No. 1 Shuaifuyuan, Wangfujing Street, Beijing, 100730 China

**Keywords:** miR-10a-5p, TFAP2C, PDAC, Chemoresistance, Prognosis

## Abstract

**Background:**

By regulating target genes, microRNAs play essential roles in carcinogenesis and drug resistance in human pancreatic ductal adenocarcinoma (PDAC). Previous studies have shown that microRNA-10a-5p (miR-10a-5p) is overexpressed in PDAC and acts as an oncogene to promote the metastatic behavior of PDAC cells. However, the role of miR-10a-5p in PDAC chemoresistance remains unclear.

**Methods:**

The effects of miR-10a-5p on biological behaviors were analyzed. MiR-10a-5p and TFAP2C levels in tissues were detected, and the clinical value was evaluated.

**Results:**

We found that miR-10a-5p is up-regulated in gemcitabine-resistant PDAC cells and enhances PDAC cell gemcitabine resistance in vitro and vivo. Meanwhile, we also determined that miR-10a-5p promotes the migratory and invasive ability of PDAC cells. Next, we confirmed that transcription factor activating protein 2 gamma (TFAP2C) is a target of miR-10a-5p, and TFAP2C overexpression resensitizes PDAC cells to gemcitabine, which is initiated by miR-10a-5p. Further studies revealed that TFAP2C also decreased PDAC cell migration and invasion capability. Finally, survival analysis demonstrated that high miR-10a-5p expression levels and low TFAP2C expression levels were both independent adverse prognostic factors in patients with PDAC.

**Conclusion:**

Together, these results indicate that miR-10a-5p/TFAP2C may be new therapeutic target and prognostic marker in PDAC.

**Electronic supplementary material:**

The online version of this article (10.1186/s13046-018-0739-x) contains supplementary material, which is available to authorized users.

## Background

Pancreatic ductal adenocarcinoma (PDAC) is the most deadly malignancy, with a overall survival rate of 7% 5 years after diagnosis [1]. The poor prognosis is due in part to the lack of diagnostic symptoms during the early stages of this disease, and approximately 80%~ 85% of patients with PDAC are not candidates for resection at the time of diagnosis. Chemotherapy, mostly gemcitabine and gemcitabine-based combinations, is commonly used to treat of these unresectable PDAC patients. However, owing to the intrinsic or external factors that promote chemotherapy resistance, among the gemcitabine-treated patients with metastatic disease, the 1-year survival rate is only 17 to 23% [2]. Among patients with metastatic PDAC, 34.5% of these patients present with disease progression during gemcitabine treatment [3]. Thus, a better understanding and quicker ability to recognize mechanisms of gemcitabine chemoresistance may provide new treatments and better drug selection strategies for improving the prognosis of this lethal disease.

MicroRNAs (miRNAs) are a class of small non-coding RNAs that are composed of 19~ 25 nucleotides. They negatively regulate genes at the post-transcriptional level by binding to the 3′ or 5′ untranslated region (UTR) of target mRNAs. In recent years, many studies have revealed that miRNA dysregulation is involved in PDAC carcinogenesis and drug resistance by serving as oncogenes or tumor suppressors [4]. Indeed, recent evidence has shown that dysregulated miRNAs participate in PDAC pathogenesis, including cell proliferation, apoptosis, differentiation, invasion, migration, epithelial-mesenchymal transition (EMT), angiogenesis, etc. In addition, several studies have demonstrated that some miRNAs are critical contributors to drug resistance by regulating the above crucial processes and are important determinants of anticancer therapy efficacy in PDAC. Thus, a better understanding of the biology and underlying mechanisms of drug resistance-related miRNAs may provide potential therapeutic targets for improving PDAC prognosis.

Previously, miR-10a-5p has been reported to be overexpressed and to act as an important mediator of metastasis formation in PDAC [5, 6]. However, the essential role and underlying mechanism of miR-10a-5p in PDAC chemoresistance remain unclear. In our previous study, we identified a panel of dysregulated miRNAs associated with drug resistance in PDAC via miRNA microarray analysis of the established gemcitabine-resistant PDAC cell line AsPC-1-Gem (data unpublished). Among these miRNAs, miR-10a-5p had a high expression level (≥ 5-fold change). Thus, we presumed that miR-10a-5p might be involved in drug resistance development in PDAC. We then found that miR-10a-5p was up-regulated in the gemcitabine-resistant PDAC cells AsPC-1-Gem and enhanced PDAC cells resistance to gemcitabine in vitro and vivo. In addition, we also found that miR-10a-5p promoted the migratory and invasive ability of PDAC cells by activating the EMT signaling pathway. Next, we confirmed that TFAP2C is a direct target gene of miR-10a-5p. Consistently, TFAP2C overexpression resensitized PDAC cells to gemcitabine, which was triggered by miR-10a-5p. Further studies revealed that TFAP2C also decreased the migration and invasion capability of PDAC cells. Finally, survival analysis revealed that high miR-10a-5p expression levels and low TFAP2C expression levels were both independent adverse prognostic indicators in patients with PDAC. Therefore, these results together indicate that miR-10a-5p/TFAP2C may be new therapeutic targets and prognostic markers in PDAC.

## Methods

### Cell lines, culture and transfection

The human pancreatic ductal adenocarcinoma (PDAC) cell lines AsPC-1, BxPC-3, MiaPaCa-2, PANC-1, Su86.86 and T3M4 cells were donated by Dr. Freiss H. (University of Heidelberg, Heidelberg, Germany). The 293A cell line was purchased from the Cell Resource Center, Institute of Basic Medical Sciences, Chinese Academy of Medical Sciences & Peking Union Medical College (IBMS, CAMS/PUMC). The AsPC-1, BxPC-3 and Su86.86 cell lines were maintained in RPMI 1640 (HyClone Logan, UT, USA). The 293A, MiaPaCa-2, PANC-1 and T3M4 cells were maintained in Dulbecco’s modified Eagle’s medium (DMEM, Logan, UT, USA). All media were supplemented with 10% fetal bovine serum (FBS, HyClone) at 37 °C with 5% CO_2_. AsPC-1-Gem cells are gemcitabine-resistant AsPC-1 cells and were obtained by researchers in our lab gradually increasing the gemcitabine doses. Gemcitabine (750 ng/ml) was added into the medium to maintain the resistant AsPC-1-Gem cell phenotype for a long time. Gemcitabine was removed 1 month before the cells were used experimentally.

T3M4, Su86.86 and AsPC-1 cells were chosen for further studies (for details, see the Results), and were transfected with 50-100 nM oligonucleotides with Lipofectamine 2000 (Invitrogen, Carlsbad, CA, USA) in 6-well plates (5 × 10^5^ cells/well). All the steps were carried out according to the protocol provided by the Lipofectamine 2000 manufacturer. The miR-10a-5p mimics, miR-10a-5p inhibitor and matched controls were synthesized by Genepharma (Shanghai, China). All the oligonucleotides used are shown in Table 1.

**Table 1:** Correlations of miR-10a-5p and TFAP2C levels in tissues and clinicopathological parameters

Variables	miR-10a-5p expression			TFAP2C expression		
	Low group	High group	*P* value	Low group	High group	*P* value
	(*n* = 35)	(*n* = 55)		(*n* = 44)	(*n* = 46)	
Gender			0.270			0.670
Male	24	30		28	27	
Female	11	25		16	19	
Age(years old)			0.380			0.831
<65	23	30		25	28	
≥ 65	12	25		19	18	
Locations			0.532			0.372
Head	23	37		27	33	
Body-tail	12	18		17	13	
Perineuronal invasion			1.000			0.019
No	20	31		19	32	
Yes	15	24		25	14	
Tumor staging			0.790			0.426
T1/T2	29	44		34	39	
T3/T4	6	11		10	7	
Lymph node staging			0.519			0.832
N0	23	31		27	27	
N1	12	24		17	19	
TNM staging			0.084			0.505
I	21	22		22	21	
II	14	33		22	25	
Diabetes			0.399			0.169
No	27	47		39	35	
Yes	8	8		5	11	

### RNA extraction and real-time PCR (qRT–PCR)

PDAC cells were transfected into 6-well plates (5 × 10^5^ cells/well) for 48 h. Total RNA was extracted using TRIzol reagent (Invitrogen, Carlsbad, CA). The RNA quality was evaluated using a NanoDrop ND-1000 spectrophotometer (NanoDrop Technologies, USA) at 260- and 280-nm (A260/280) wavelengths. Complementary DNA was synthesized by TaqMan MicroRNA RT Kit (Applied Biosystems) and the reverse transcription kit (Promega, Madison, WI). Quantitative RT–PCR (qRT-PCR) was performed using TaqMan MicroRNA Assays (Applied Biosystems) and SYBR Green Master Mix (Takara, Japan). U6 RNA and GAPDH were chosen as internal controls for mRNA and miRNA detection, respectively. The relative expression of miR-10a-5p and mRNAs was calculated through the 2^-ΔΔCT^ method. All the primers used are shown in Table 1.

### Cell proliferation and growth inhibition assay

At 24 h after transfection, PDAC cells were plated into 96-well culture plates (1000 cells/well) for cell proliferation assays. All the plates were cultured at 37 °C with 5% CO_2_. For cell proliferation assays, 10 μL/well cell count kit (CCK-8) reagent was added at 0, 24, 48 and 72 h after plating. After an additional 2.5-h incubation with CCK-8 reagent at 37 °C, the optical density (OD) at the 450-nm wavelength (OD450) was measured using a microplate reader (Wellscan MK3, Thermo/Labsystems, Finland). OD630 served as a reference, and the OD in the blank well was used as the base level. For growth inhibition assays, 4000 cells/well were plated into 96-well culture plates at 24 h after transfection. After incubation for 4-6 h for cell adherence, a gemcitabine (Eli Lilly and Company) concentration gradient from 100 nM to 1 mM or control PBS buffer was added into each well. Cell count kit (CCK-8) reagent (10 μL/well) was added after an additional 48-h incubation at 37 °C. Then, the inhibition rate was calculated as follows: *OD*_*sample*_ = *OD*450 − *OD*630, $\mathrm{Inhibition}\ \mathrm{rate}=1-\frac{OD_{\mathrm{Gem}}-{OD}_{\mathrm{blank}}}{OD_{\mathrm{PBS}}-{OD}_{\mathrm{blank}}}$.

### Apoptosis assay

PDAC cells were transfected into 6-well plates and treated with 10 μM gemcitabine 24 h later. After treating for 48 h, the cells were collected and resuspended in binding buffer. Next, the cells were stained with Annexin V-FITC and propidium iodide (PI) (Beyotime, China) according to the manufacturer’s instructions. The analysis was carried out using flow cytometry (FACScan; BD Biosciences, USA).

### In vitro transwell assays

Transwell assays were performed in triplicate using transwell migration chambers (8-μm pore size; Corning, USA) for cell migration and invasion experiments. For invasion assays, wells were coated with diluted ECM solution (Sigma-Aldrich, Shanghai, China). Cells transfected with miR-10a-5p mimics, inhibitors or paired control oligonucleotides were transferred to the top of the upper chambers or to the ECM gel in the serum-free culture. After culturing for 48 h, medium containing 10% FBS was added to the lower chambers. The cells that migrated or invaded into the lower surface after 48 h of incubation were fixed in 90% ethyl alcohol and stained with hematoxylin-eosin for counting. The number of cells in the chamber were counted in five random visual fields under a microscope at 100× magnification. The average number of cells counted in the five fields was used as the final result. All experiments were performed three times.

### Western blotting

After transfection for 48 h, total cellular protein was extracted with RIPA lysis buffer (Applygen, Beijing). Total protein (100 μg) was separated on SDS-PAGE gel and then transferred to a PVDF membrane (Millipore, Billerica, MA). The membrane was probed with primary antibodies (1:1000, Danvers, MA) overnight at 4 °C after blocking with 5% non-fat milk at room temperature for 1 hour. The next day, the membrane was incubated with horseradish peroxidase-conjugated secondary antibody (1:3000, Applygen, Beijing) at room temperature for 1 hour. Protein bands were detected by ECL reagents (Millipore, Billerica, MA).

### Animal experiments

AsPC-1 cells stably transfected with miR-10a-5p lentiviral vectors or control (Lv-AsPC-1-miR-10a-5p or Lv-AsPC-1-NC) were injected subcutaneously into the right flank of 6-week-old female BALB/c mice (Shanghai, Chinese Academy of Sciences, China) (5 × 10^6^ cells in 250 μl of PBS per mouse). Each experimental group included five mice. Gemcitabine (50 mg/kg) was administered by intraperitoneal injections 1 week after tumor formation (tumor size between 100 to 200 mm^3^), followed by periodic booster shots every 3 days for 4 weeks. Two perpendicular tumor diameters was measured once a week using a caliper. Tumor volume (mm^3^) was calculated: volume (mm^3^) = 1/2 × length × width^2^. All tumor-bearing mice were euthanized on the 35^th^ day.

### Fluorescent reporter assay

The pmirGLO dual luciferase miRNA target expression vector (Promega, E1330) was used to assess miR-10a-5p regulation of miRNA target sites. Wild-type (WT) or mutant (MUT) miR-10a-5p binding site sequences in the 3’-UTR of TFAP2C were synthesized by Invitrogen and cloned into pmirGLO vectors at the 3′-end of the firefly luciferase gene. Vectors and miR-10a-5p mimics or mimics controls were co-transfected into 293A cells with Lipofectamine 2000 reagent in 12-well plates. Forty-eight hours later, luciferase activity was evaluated using a Dual-Luciferase Reporter Assay system (Promega). Renilla luciferase (hRlucneo) served as the control reporter for normalization.

### Patients and sample collection

Formalin-fixed, paraffin-embedded PDAC tissues (*n* = 90) and matched tumor-adjacent tissues (*n* = 90) were collected from PUMCH and were made tissue microarrays. None of the patients received neoadjuvant therapy before surgical resection. PDAC was diagnosed and staged by pathological examination by two pathologists independently. Patients with TNM stages later than stage II were not included in this research. Follow-up data were obtained from medical records and follow-up. The end-point was overall survival (OS). Survival time was defined according to the time between the date of death or the last follow-up date and the operation date.

### Pancreatic tissue collection and in situ hybridization (ISH)

The miRCURY LNA detection probe (18017-15; Exiqon, Vedbaek, Denmark) was used to detect the miR-10a-5p expression levels in tissue microarrays. After deparaffinization in xylene and rehydration with graded alcohol washes, the slides were fixed in 4% paraformaldehyde for 20 min. After washing in PBS three times, the slides were incubated with 15 μg/ml proteinase K for 15 min at room temperature. The slides were then washed in PBS and fixed in 4% paraformaldehyde for 15 min. After rinsing in PBS, the slides were prehybridized in hybridization buffer for 1 h at 50 °C and then hybridized in probe-containing hybridization buffer overnight at 4 °C. The following day, the slides were washed stringently at 50 °C for 20 min, followed by blocking in a blocking solution for 1 h at room temperature. Finally, the slides were placed in blocking solution containing alkaline phosphatase-conjugated anti-DIG Fab fragment overnight at 4 °C again. The colorimetric detection reaction was carried out using the NBT/BCIP kit (Thermo Fisher Scientific). The slides were scored according to the staining intensity and percentage of positive cells. The scoring for stain intensity was as follows: none (0 points), weak staining (1 point), intermediate staining (2 points), and strong staining (3 points). The scoring for the positive cell percentage was as follows: absent (0 points), 1–24% positive cells (1 point), 25–49% (2 points), 50–74% (3 points), and 75–100% (4 points). The final score was calculated by multiplying the above two scores. MiR-10a-5p expression was considered to be low if the final score was less than the median value and high if the final score was the median value or above.

### Immunohistochemistry (IHC)

Rabbit anti-human TFAP2C polyclonal antibodies (ab218107, Abcam) were used for staining. Slides were deparaffinized in xylene and rehydrated in a graded alcohol series. After washed with PBS, endogenous peroxidase activity of the slides was blocked with 3% H_2_O_2_ for 10 min. Incubate the slides in 0.1% trypsin for antigen retrieval and heating them in a microwave oven. The slides were incubated with primary antibody (1:200) overnight at 4 °C then. After washed three times with PBS, the slides were incubated with horseradish peroxidase (HRP)-conjugated secondary antibody for 30 min at 37 °C. Diaminobenzidine served as a chromogen. The slides were then counterstained with hematoxylin. Nonimmune rabbit serum served as the negative control. TFAP2C expression levels were scored according to the staining intensity and percentage of positive cells, either. The scoring for stain intensity was as follows: none (0 points), weak staining (1 point), intermediate staining (2 points) and strong staining (3 points). The scoring for the positive cell percentage was as follows: absent (0 points), 1–24% of the cells (1 point), 25–49% of the cells (2 points), 50–74% of the cells (3 points), and 75–100% of the cells (4 points). The final score was calculated by multiplying the above two scores. TFAP2C expression was considered to be low if the final score was less than the median value and was considered high if the final score was the median value or above.

Sequence of the primers used in this study.

**Table:** 

Oligonucleotides/Primer	Sequence (5′–3′)
MiR-10a-5p mimics sense	CAAAUUCGGAUCUACAGGGUAUU
MiR-10a-5p mimics antisense	UACCCUGUAGAUCCGAAUUUGUG
Mimics control sense	UUCUCCGAACGUGUCACGUTT
Mimics control antisense	UACCCUGUAGAUCCGAAUUUGUG
MiR-10a-5p inhibitor	CACAAAUUCGGAUCUACAGGGUA
Inhibitor control	CAGUACUUUUGUGUAGUACAA
TFAP2C sense	ATCTTGGAGGACGAAATGAGAT
TFAP2C antisense	CAGATGGCCTGGCTGCCAA
GAPDH sense	CGGAGTCAACGGATTTGGTCGTAT
GAPDN antisense	AGCCTTCTCCATGGTGGTGAAGAC

## Results

### MiR-10a-5p enhances gemcitabine resistance in vitro and in vivo

**Fig. 1:**
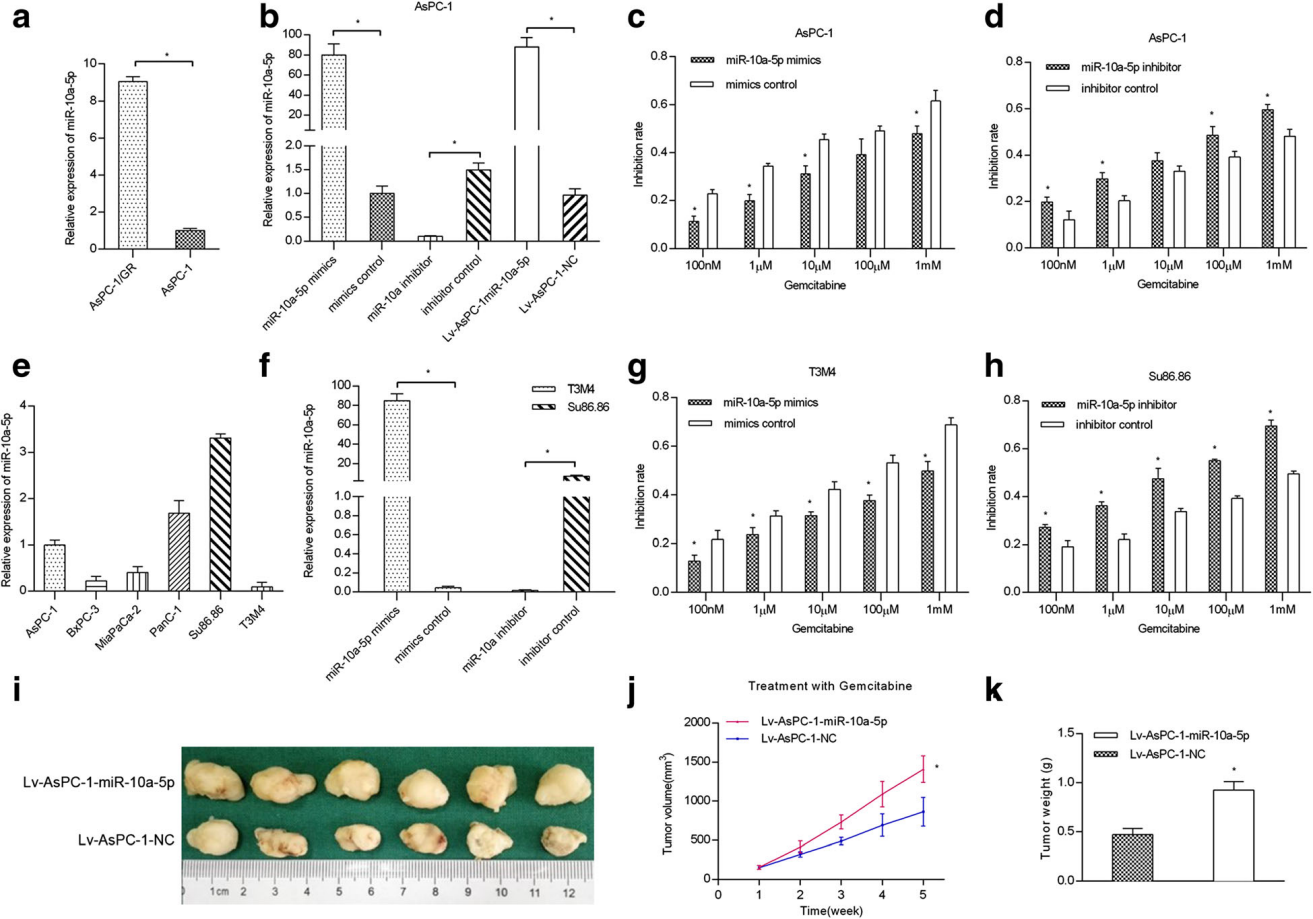
MiR-10a-5p enhances gemcitabine resistance in vitro and vivo. **a** MiR-10a-5p was significantly up-regulated in AsPC-1-Gem cells (AsPC-1/GR), as determined using quantitative RT–PCR; **b** transient and stable miR-10a-5p transfection efficiency in the AsPC-1 cell line; **c** miR-10a-5p mimics significantly increased gemcitabine resistance in AsPC-1 cells; **d** miR-10a-5p inhibitor significantly decreased gemcitabine resistance in AsPC-1 cells; **e** miR-10a-5p levels in six PDAC cell lines; **f** transfection efficiency of miR-10a-5p in T3M4 and Su86.86 cell lines; **g** miR-10a-5p mimics significantly increased gemcitabine resistance in T3M4 cells; **h** miR-10a-5p inhibitor significantly decreased gemcitabine resistance in Su86.86 cells; **i** pictures of tumors from nude mice 4 weeks after the gemcitabine treatment; **j** tumor growth in nude mice after the gemcitabine injections; **k** tumors with high miR-10a-5p levels were significantly larger than control tumors 4 weeks after treating with gemcitabine. The data are presented as the means ± SD. (Student’s t-test; *, *P* < 0.05)

To determine if miR-10a-5p is potentially associated with gemcitabine resistance in PDAC, we first performed quantitative RT–PCR to validate miR-10a-5p expression in the previously established gemcitabine-resistant PDAC cell line AsPC-1-Gem. The data revealed that the miR-10a-5p expression level was significantly up-regulated in the AsPC-1-Gem cells (Fig. 1a). We then tested the miR-10a-5p expression level in the six PDAC cell lines and found that T3M4 cells had the lowest expression level, while Su86.86 cells had the highest expression level (Fig. 1e); therefore, these two cell lines were chosen for further investigation (Fig. 1e and f). Next, growth inhibition assays were used to test the chemosensitivity of PDAC cells to gemcitabine in vitro. As shown in Fig. 1c and g, the miR-10a-5p mimics transfection decreased the inhibition rate of PDAC cells, which meant that miR-10a-5p significantly increased cell gemcitabine resistance. In contrast, compared with cells transfected with inhibitor controls, PDAC cells transfected with miR-10a-5p inhibitors were more sensitive to gemcitabine (Fig. 1d and h). To further determine the effects of miR-10a-5p on chemosensitivity in vivo, we established AsPC-1 cell lines that stably overexpressed miR-10a-5p, and we subcutaneously injected these cells into nude mice treated with gemcitabine (Fig. 1i). The in vivo results revealed that in mice treated with gemcitabine, the tumors generated from the Lv-AsPC-1- miR-10a-5p cells grew significantly faster than from Lv-AsPC-1-NC (Fig. 1j). In addition, tumor weight was significantly increased in the PDAC cells overexpressing miR-10a-5p (Fig. 1k). Thus, these results suggest that miR-10a-5p decreases gemcitabine-induced cytotoxicity in vitro and in vivo.

### MiR-10a-5p promotes PDAC cell migration and invasion

**Fig. 2:**
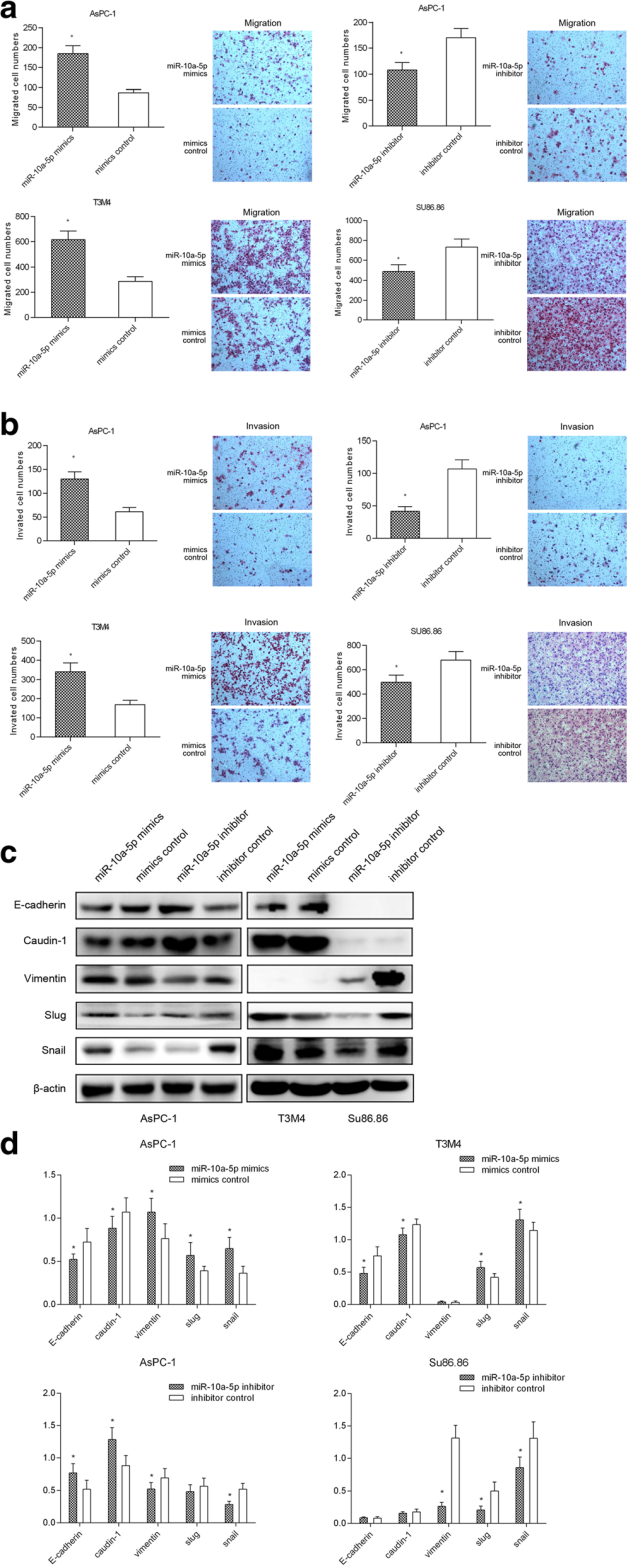
MiR-10a-5p promotes PDAC cell migration and invasion. **a** MiR-10a-5p overexpression promoted T3M4 and AsPC-1 cell migration, while miR-10a-5p knockdown decreased Su86.86 and AsPC-1 cell migration; **b** miR-10a-5p overexpression promoted T3M4 and AsPC-1 cell invasion, while miR-10a-5p knockdown decreased Su86.86 and AsPC-1 cell invasion; **c** miR-10a-5p expression up-regulation decreased E-cadherin and Caudin-1 protein levels while increasing Vimentin, Slug and Snail levels, as determined by using western blotting. **d** The relative intensity of the grayscale band values revealed the changes with miR-10a-5p overexpression or knockdown in PDAC cells; the data are presented as the means ± SD (Student’s t-test; *, *P* < 0.05)

To evaluate the oncogenic properties of miR-10a-5p in PDAC cells, we also assessed the effects of miR-10a-5p on cell proliferation, apoptosis, cell cycle, migration and invasion by either miR-10a-5p gain- or loss-of-function assays. As shown in Fig. 2a, compared with control AsPC-1 and T3M4 cells, the AsPC-1 and T3M4 cells with miR-10a-5p overexpression by mimics transfection presented with a significantly increased migration capacity, while miR-10a-5p knockdown by transfection inhibitors significantly decreased the number of migrated AsPC-1 and Su86.86 cells. Meanwhile, the invasion assay resulted in similar results (Fig. 2b). However, miR-10a-5p exerted no effects on cell proliferation, apoptosis and cell cycle via either miR-10a-5p gain- or loss-of-function assays in PDAC cells (Additional file 1: Figure S1). To further determine the molecular mechanisms underlying the migratory and invasive properties of miR-10a-5p observed in the PDAC cells above, we used western blotting to investigate the expression levels of EMT-related genes potentially regulated by miR-10a-5p. As shown in Fig. 2c, miR-10a-5p overexpression in AsPC-1 and T3M4 cells increased the Vimentin, Slug and Snail levels, while the E-cadherin and Caudin-1 expression levels was decreased. By contrast, opposing effects were observed in AsPC-1 and Su86.86 cells with miR-10a-5p knockdown. In summary, these data indicated that miR-10a-5p promoted the migratory and invasive ability of PDAC cells.

### MiR-10a-5p targets TFAP2C to increase gemcitabine resistance

**Fig. 3:**
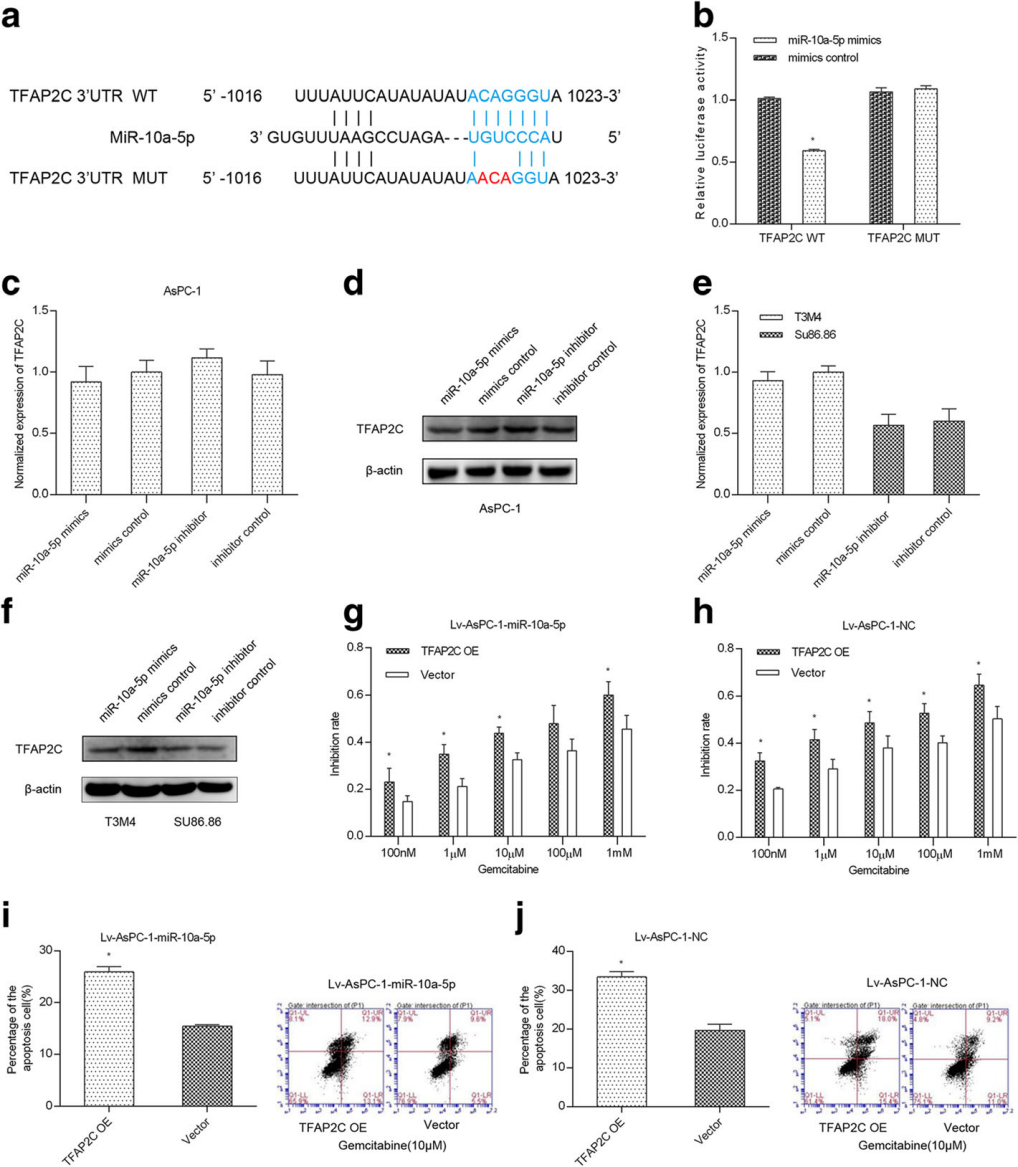
MiR-10a-5p targets TFAP2C to increase gemcitabine resistance. **a** Luciferase reporter constructs containing downstream wild-type (WT) and mutated (MUT) binding sequences in TFAP2C; **b** the luciferase activity of the WT and MUT groups suggests that TFAP2C is a direct target of miR-10a-5p; **c** miR-10a-5p did not change the TFAP2C mRNA levels in the AsPC-1 cell line; **d** miR-10a-5p expression up-regulation decreased the TFAP2C protein level in the AsPC-1 cell line; **e** miR-10a-5p expression did not change the TFAP2C mRNA levels in the T3M4 and Su86.86 cell lines; **f** miR-10a-5p expression up-regulation decreased the TFAP2C protein level in the T3M4 and Su86.86 cell lines; **g** TFAP2C expression up-regulation in AsPC-1 cells stably overexpressing miR-10a-5p significantly increased gemcitabine chemosensitivity; **h** TFAP2C expression up-regulation in AsPC-1 cells stably overexpressing mimics control also significantly increased gemcitabine chemosensitivity; **i** TFAP2C expression up-regulation in AsPC-1 cells stably overexpressing miR-10a-5p significantly increased gemcitabine-induced cell apoptosis; **j** TFAP2C up-regulation in AsPC-1 cells stably overexpressing mimics control significantly increased gemcitabine-induced cell apoptosis. The data are presented as the means ± SD (Student’s t-test; *, *P* < 0.05)

TFAP2C was predicted as a potential target of miR-10a-5p using the bioinformatics database (TargetScan; http://www.targetscan.org/). We then performed dual-luciferase reporter assays for verification. Luciferase reporter constructs containing either the wildtype (WT vectors) or mutated (MUT) TFAP2C binding sequences downstream of the firefly luciferase gene were generated (Fig. 3a). The 293A cells were co-transfected with reporter vectors and mimics or mimics control. Luciferase activity was evaluated. Luciferase activity was significantly decreased after miR-10a-5p mimics co-transfection with WT vectors compared with the activity in cells co-transfected with mimics and MUT vectors, with mimics control and WT vectors, or with mimics control and MUT vectors (Fig. 3b). These results suggest that TFAP2C is a direct target of miR-10a-5p.

To further confirm whether TFAP2C is regulated by miR-10a-5p, we used qRT–PCR and western blotting to verify the mRNA and protein level changes, respectively, in respect to changes in the miR-10a-5p level. The results revealed that miR-10a-5p did not affect TFAP2C mRNA levels in AsPC-1, T3M4 and Su86.86 cells (Fig. 3c and e). However, miR-10a-5p up-regulation decreased the TFAP2C protein level in the AsPC-1 and T3M4 cell lines (Fig. 3d and f). In contrast, miR-10a-5p inhibition induced the opposite effect in AsPC-1 and Su86.86 cell lines (Fig. 3d and f).

We then investigated whether miR-10a-5p-enhanced gemcitabine resistance was dependent on TFAP2C expression. The CCK-8 assay was performed to detect the gemcitabine chemosensitivity of Lv-AsPC-1-miR-10a-5p and Lv-AsPC-1-NC cells overexpressing TFAP2C. The Lv-AsPC-1-miR-10a-5p and Lv-AsPC-1-NC cells transfected with the TFAP2C vector were more sensitive to gemcitabine than the cells transfected with negative controls (Fig. 3g and h). Furthermore, compared with the control group, the apoptosis assessment using flow cytometry revealed that cells overexpressing TFAP2C exhibited an increased apoptosis rate after incubating with gemcitabine for 48 h (Fig. 3i and j). Thus, all these results revealed that miR-10a-5p targets TFAP2C to increase gemcitabine resistance.

### TFAP2C suppresses PDAC cell migration and invasion

**Fig. 4:**
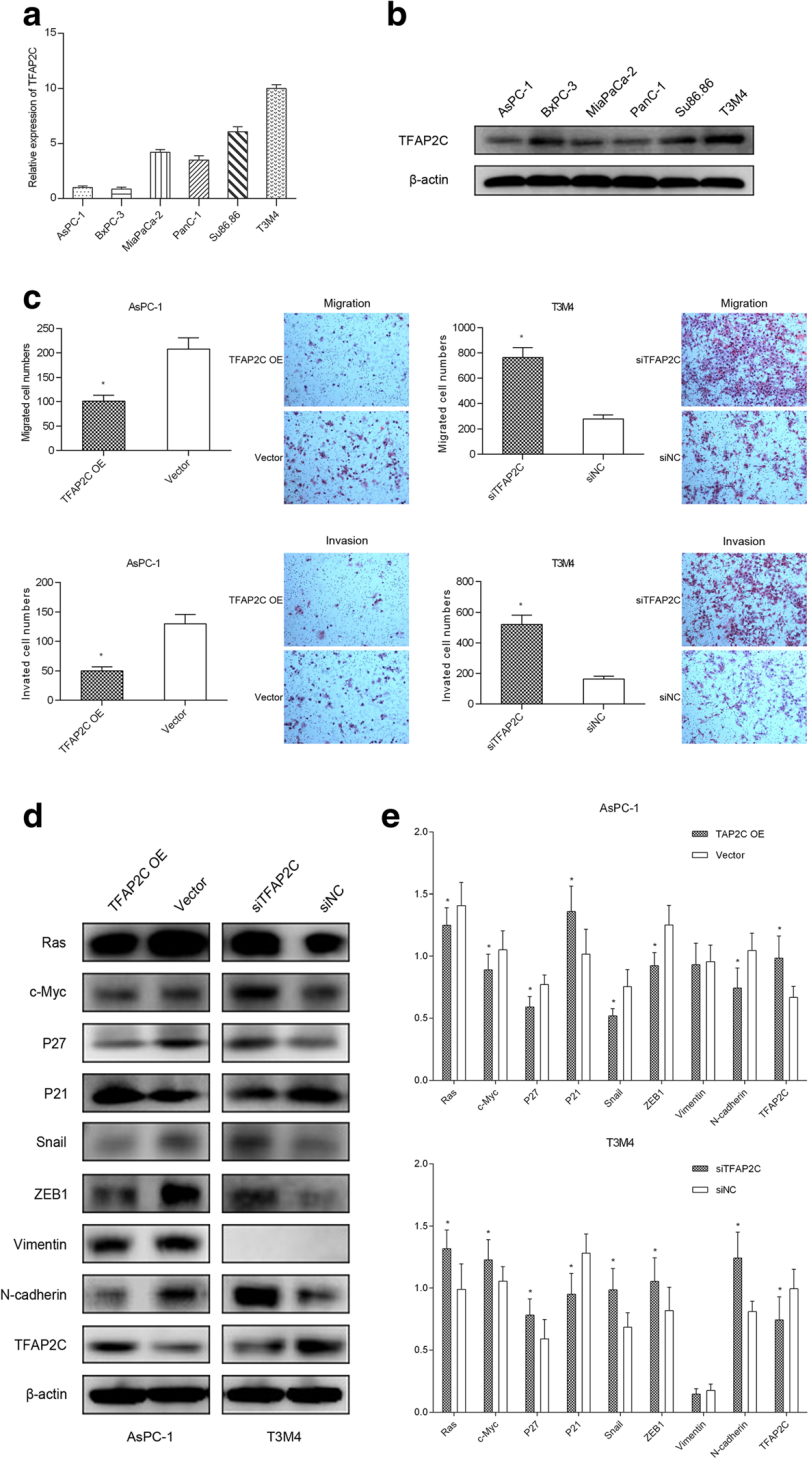
TFAP2C suppresses PDAC cell migration and invasion. **a** TFAP2C mRNA levels in six pancreatic cell lines; **b** TFAP2C protein levels in six pancreatic cell lines; **c** TFAP2C overexpression decreased cell migration and invasion, while TFAP2C knockdown using siRNA significantly increased cell migration and invasion; **d** TFAP2C negatively regulated Ras, c-Myc, p27, Snail, ZEB1 and N-Cadherin protein levels and up-regulated the p21 level in AsPC-1 and T3M4 cells. **e** The relative intensity of the grayscale band values revealed the changes with TFAP2C overexpression or knockdown in PDAC cells. The data are presented as the means ± SD (Student’s t-test; *, *P* < 0.05)

The above results demonstrated that miR-10a-5p targeted TFAP2C to promote gemcitabine resistance in PDAC, but the role of TFAP2C in PDAC remains unclear. Thus, by either gain- or loss-of-function assays, we examined the potential oncogenic role of TFAP2C on cell proliferation, migration and invasion and the cell cycle in PDAC cells. As shown in Fig. 4a and b, we first examined the TFAP2C expression levels in six PDAC cell lines and found that T3M4 had the TFAP2C highest protein level, while AsPC-1 had the lowest protein level; therefore, these two cell lines were chosen for further investigation. Data showed that TFAP2C overexpression attenuated AsPC-1 cell migration and invasion, as measured by transwell assays (Fig. 4c). In contrast, TFAP2C knockdown in T3M4 cells promoted tumor cell migration and invasion (Fig. 4c). However, TFAP2C exerted no effects on cell proliferation or the cell cycle via either gain- or loss-of-function assays in PDAC cells (Additional file 2: Figure S2). To further determine the underlying molecular mechanisms of TFAP2C in the PDAC cells observed above, we used western blotting to investigate the expression levels of related genes potentially regulated by TFAP2C. As shown in Fig. 4d, TFAP2C overexpression in AsPC-1 cells led to decreased Ras, c-Myc, p27, Snail, ZEB1 and N-Cadherin levels, while the p21 expression levels were increased. In contrast, the opposite effects were observed in T3M4 cells with TFAP2C knocked down. Thus, these data indicated that TFAP2C attenuated the migratory and invasive ability of PDAC cells.

### High miR-10a-5p level is associated with poor prognosis

**Fig. 5:**
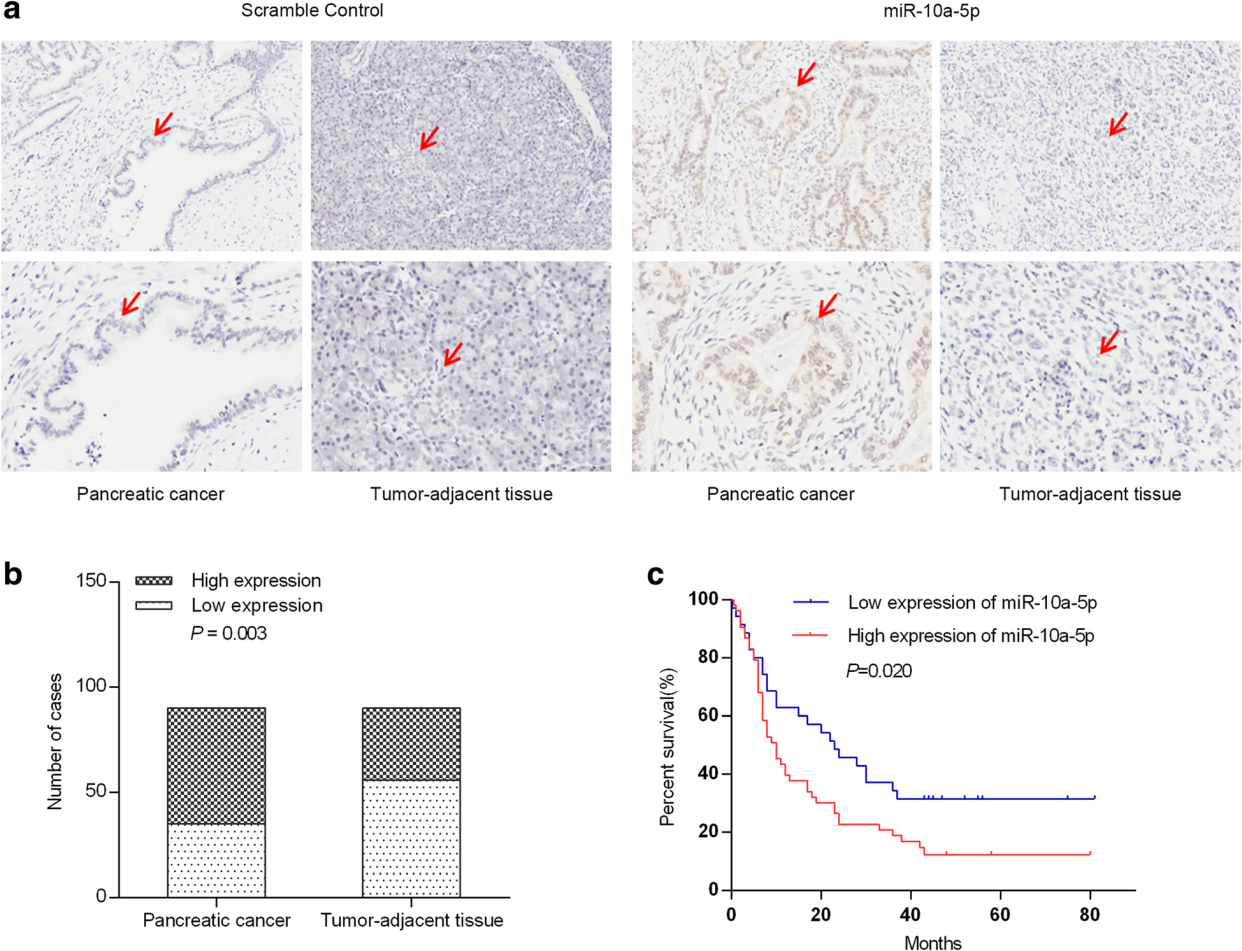
MiR-10a-5p is up-regulated in PDAC. **a** The miR-10a-5p expression level in 90 PDAC tissue samples and matched tumor-adjacent tissues evaluated by in situ hybridization (ISH). Left pictures in each row are negative ISH controls; **b** the miR-10a-5p expression levels in 90 PDAC tissue samples and matched tumor-adjacent tissues were analyzed using the Pearson χ2 test; **c** Kaplan-Meier survival analysis revealed that a high miR-10a-5p expression level in tumors was significantly associated with reduced survival in patients with PDAC

We next evaluated by ISH the miR-10a-5p expression levels in 90 PDAC tissue samples and matched tumor-adjacent tissue samples (Fig. 5a). Tissue samples were scored for high or low miR-10a-5p expression as described in Materials and Methods. Among the 90 PDAC samples, 35 samples presented with low miR-10a-5p expression, and 55 samples presented with high expression. Among the matched tumor-adjacent tissues, 56 presented with low miR-10a-5p expression, whereas 34 presented with high expression. MiR-10a-5p expression was significantly increased in PDAC tissues compared with tumor-adjacent tissues (*P* = 0.003) (Fig. 5b).

We assessed the correlation between miR-10a-5p levels and clinicopathological parameters (Table 1). The mean follow-up was 22.38 months (range 1–81 months). No correlation was observed between the miR-10a-5p levels and clinicopathological parameters. Survival analysis was also carried out (Table 2). Univariate survival analysis indicated that miR-10a-5p expression levels, lymph node staging and TNM staging were the potential prognostic factors in PDAC (*P* = 0.020) (Fig. 5c). Multivariate analysis demonstrated that miR-10a-5p expression (high) was an independent adverse prognostic factor (*P* = 0.000, hazard ratio [HR] = 2.878, 95% confidence interval [CI]: 1.614-5.131).

**Table 2:** Univariate and multivariable analyses of factors predictive of poor overall survival in pancreatic cancer patients

Variables	Univariate analysis			Multivariate analysis		
	Overall survival	95% confidence interval	*P* value	Hazard ratio	95% confidence interval	*P* value
	(Median ± SE,months)					
Gender			0.342	0.675	0.397-1.148	0.147
Male	23.685 ± 3.702	16.430-30.941				
Female	24.640 ± 3.679	17.430-31.850				
Age(years old)			0.653	0.999	0.616-1.622	0.997
<65	26.863 ± 3.838	19.339-34.386				
≥ 65	20.155 ± 3.405	13.482-26.828				
Locations			0.576	1.109	0.659-1.868	0.696
Head	27.543 ± 3.832	20.032-35.054				
Body-tail	22.933 ± 4.641	13.837-32.029				
Perineuronal invasion			0.645	1.155	0.668-1.998	0.605
No	27.687 ± 4.269	19.320-36.054				
Yes	24.122 ± 4.181	15.928-32.316				
Tumor staging			0.173	0.568	0.185-1.742	0.322
T1/T2	28.275 ± 3.560	21.298-35.253				
T3/T4	17.824 ± 4.633	8.743-26.904				
Lymph node staging			0.005	0.631	0.194-2.049	0.443
N0	33.336 ± 4.398	24.715-41.956				
N1	14.520 ± 2.408	9.801-19.240				
TNM staging			0.000	4.501	1.253-16.161	0.021
I	40.521 ± 5.492	29.757-51.286				
II	15.706 ± 2.506	10.795-20.618				
Diabetes			0.717	1.808	0.895-3.652	0.099
No	25.790 ± 3.315	19.294-32.287				
Yes	22.350 ± 5.087	12.379-32.321				
MiR-10a expression			0.020	2.878	1.614-5.131	0.000
Low	35.489 ± 5.446	24.814-46.164				
High	20.195 ± 3.316	13.697-26.694				
TFAP2 expression			0.039	0.46	0.261-0.809	0.007
Low	19.367 ± 3.568	12.373-26.360				
High	32.159 ± 4.539	23.263-41.055				

### Low TFAP2C expression is associated with poor prognosis

**Fig. 6:**
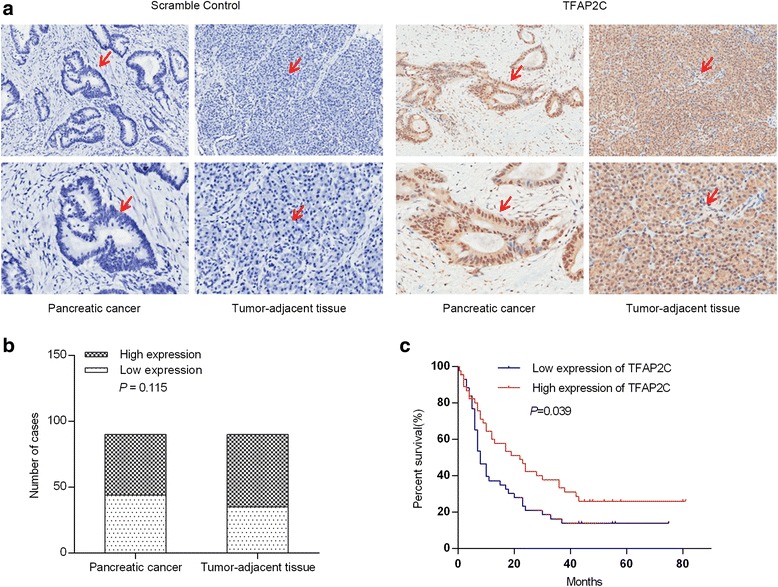
Low TFAP2C expression is associated with poor prognosis. **a** The TFAP2C expression levels in 90 PDAC tissue samples and matched tumor-adjacent tissues evaluated by immunohistochemistry. Left pictures in each row are negative immunohistochemistry controls. **b** TFAP2C expression had a downward trend in PDAC tissues compared with tumor-adjacent tissues. **c** Kaplan-Meier survival analysis revealed that low TFAP2C expression levels in tumors were significantly associated with reduced survival in PDAC patients

We evaluated the TFAP2C expression levels in 90 PDAC tissue samples and matched tumor-adjacent tissues by IHC staining (Fig. 6a). The IHC staining results revealed that TFAP2C was mainly located in the nucleus. The tissue samples were scored for high or low TFAP2C expression as described in Materials and Methods. Among the 90 PDAC samples, 44 presented with low TFAP2C expression, and 46 had high expression. Among the matched tumor-adjacent tissues, 35 presented with low TFAP2C expression, whereas 55 had high expression. TFAP2C expression trended downward in PDAC tissues compared with tumor-adjacent tissues (*P* = 0.1147) (Fig. 6b).

We also assessed the correlation between TFAP2C levels and clinicopathological parameters in ninety patients (Table 1). TFAP2C was associated with perineuronal invasion. No other correlation was observed between the TFAP2C levels and clinicopathological parameters. Survival analysis was also carried out (Table 2). Univariate survival analysis indicated that the TFAP2C expression level was also a potential prognostic factor in PDAC (*P* = 0.039) (Fig. 6c). Multivariate analysis demonstrated that TFAP2C expression (low) was an independent adverse prognostic factor (*P* = 0.007, hazard ratio [HR] = 0.460, 95% confidence interval [CI]: 0.261-0.809).

## Discussion

Chemoresistance is one of the main causes of poor prognosis in PDAC. Thus, investigating the mechanisms underlying chemoresistance and chemotherapy resensitization in PDAC cells is critical for PDAC treatment. In the present study, we identified that miR-10a-5p was up-regulated in gemcitabine-resistant PDAC cells and found that miR-10a-5p enhanced PDAC cell resistance to gemcitabine in vitro and vivo. In addition, miR-10a-5p promoted the migratory and invasive ability of PDAC cells though up-regulating EMT-related gene expression. Mechanistically, miR-10a-5p directly targeted TFAP2C to confer gemcitabine resistance. Meanwhile, TFAP2C acted as a tumor suppressor to decrease the PDAC cell migration and invasion capability and negatively modulated EMT-associated gene expression. We also demonstrated that high miR-10a-5p expression and low TFAP2C expression are significantly associated with poor prognosis in patients with PDAC. In this regard, our data indicated that miR-10a-5p/TFAP2C were valuable prognostic predictors of PDAC and appeared to be promising targets for PDAC therapy.

It has been reported that miR-10a-5p plays varying but important roles in multiple cancers. Wang et al. [7] found that miR-10a-5p suppresses the miR-10a-EphA4 axis, promoting cell proliferation, invasion and EMT in hepatic cell cancer. In non-small cell lung cancer (NSCLC), in vitro experiments revealed that miR-10a-5p overexpression promoted NSCLC cell proliferation, migration and invasion by directly targeting PTEN [8]. In breast cancer [9], miR-10a-5p promotes cell migration, which is positively regulated by RUNX2. In cervical cancer [10], miR-10a-5p promotes cell colony formation, migration and invasion by targeting CHL1. However, in other studies, miR-10a-5p acts very differently. In gastric cancer, miR-10a-5p represses cell growth, migration and invasion through silencing HoxA1 [11]. In breast cancer [12], one article reported that miR-10a-5p was significantly down-regulated in malignant cells compared with normal or benign glandular cells, indicating that miR-10a-5p might act as a tumor suppressor. Regarding tumor chemosensitivity, miR-10a-5p also plays controversial roles. Studies have shown that miR-10a-5p is associated with cisplatin (DDP) resistance in lung cancer. Silencing miR-10a-5p in DDP-resistant cells increases cell chemosensitivity to DDP, induces cell apoptosis and up-regulates caspase 3/8 expression and activity [13]. However, in ER-positive breast cancer [14], Cox regression analysis revealed that increased miR-10a-5p expression is associated with a long relapse-free time following tamoxifen treatment. Our study was the first to investigate the differential miR-10a-5p expression in gemcitabine-resistant and parental cell lines. We found that miR-10a-5p was significantly up-regulated in gemcitabine-resistant cells and promoted PDAC cell migration and invasion in vitro. Further studies revealed that miR-10a-5p enhances gemcitabine resistance in vitro and vivo.

MiR-10a-5p has also been reported to be overexpressed in cancer cells compared with normal tissues [10, 15–17] and to be up-regulated in metastatic [16] or recurrent [9] tumor cells compared with primary cancer cells. Li et al. [18] identified a relationship between the miR-10a-5p level and both disease-free survival and OS in gastric cancer. In NSCLC [8], miR-10a-5p expression is higher in highly metastatic cells rather than in weakly metastatic cells or normal tissues, as determined by miRNA expression microarray, and relative factor analysis reveals that high miR-10a-5p expression is associated with later lymph node (N) and metastasis (M) stages. Our study used ISH to reveal that miR-10a-5p is up-regulated in PDAC tissue samples compared with matched tumor-adjacent tissues. We found no correlation between miR-10a-5p levels and clinicopathological parameters. However, univariate and multivariate survival analysis both indicated that miR-10a-5p expression (high) is an independent adverse prognostic factor in PDAC.

Recent studies on TFAP2C have mainly focused on breast cancer and lung cancer. In breast cancer, TFAP2C participates in regulating luminal-specific genes [19] and is involved in multiple cell proliferation pathways, indicating that TFAP2C is a potential drug target site [20]. TFAP2C may also be associated with breast cancer prognosis: higher TFAP2C levels correlate with poor overall survival in unique ERα(+) and endocrine therapy-treated subgroups [21], and high tissue TFAP2C expression also contributes to the failure of anti-hormone treatments [22]. In NSCLC, Kang, J. et al [23] found that TFAP2C overexpression is associated with cell tumorigenesis and cell cycle activation via the miR-183 and miR-33a pathways in vivo. Other researchers have suggested that the slug-miR-137-TFAP2C axis may provide new candidate target molecules for lung cancer therapeutics [24]. However, in our study results, TFAP2C works as a tumor suppressor and can suppress PDAC cell migration and invasion, as well as negatively regulate certain protein levels. On the other hand, silencing TFAP2C up-regulates p21 levels. Furthermore, univariate and multivariate analysis both demonstrated that low TFAP2C expression was an independent adverse prognostic factor. Our study is the first to investigate the roles of TFAP2C in PDAC and to elucidate the tumor suppresser role of this protein.

## Conclusion

In conclusion, we used quantitative RT–PCR to identify miR-10a-5p up-regulation in gemcitabine-resistant PDAC cell lines. Additional studies demonstrated that miR-10a-5p promotes PDAC cell migration and invasion. MiR-10a-5p also enhances gemcitabine resistance in vitro and in vivo by directly targeting TFAP2C. Methods that suppress TFAP2C promote cancer cell migration and invasion. Survival analysis demonstrated that a high miR-10a-5p level and low TFAP2C are both independent adverse prognostic factors in PDAC.

## Additional files

Additional file 1: **Figure S1.** MiR-10a-5p does not influence cell proliferation, cell apoptosis or the cell cycle. (A) MiR-10a-5p overexpression in the T3M4 cell line had a trend of promoting cell proliferation, but proliferation was not significantly different between the overexpressing cells and control cells. (B) MiR-10a-5p overexpression in the T3M4 cell line had a trend of decreasing gemcitabine-induced cell apoptosis, but apoptosis was not significantly different between the overexpressing cells and control cells. (C) MiR-10a-5p knockdown in the Su86.86 cell line had a trend of suppressing cell proliferation, but proliferation was not significantly different between the knockdown cells and control cells. (D) MiR-10a-5p knockdown in the Su86.86 cell line had a trend of promoting gemcitabine-induced cell apoptosis, but apoptosis was not significantly different between the knockdown cells and control cells. (E) MiR-10a-5p overexpression in the T3M4 cell line had a trend of accelerating the S-G2 transition of the cell cycle, but this transition rate was not significantly different between the overexpressing cells and control cells. (F) MiR-10a-5p knockdown in the Su86.86 cell line reduced the S-G2 transition of the cell cycle. The data are presented as the means ± SD (Student’s t-test; *, *P* < 0.05). (PNG 994 kb)

Additional file 2: **Figure S2.** TFAP2C does not influence cell proliferation or the cell cycle. (A) TFAP2C overexpression in the AsPC-1 cell line had a trend of suppressing cell proliferation, but proliferation was not significantly different between the overexpressing cells and control cells. (B) TFAP2C knockdown in the T3M4 cell line had a trend of promoting cell proliferation, but proliferation was not significantly different between the knockdown cells and control cells. (C) TFAP2C overexpression in the AsPC-1 cell line had a trend of reducing the G1-S transition of the cell cycle, but this transition rate was not significantly different between the overexpressing cells and control cells. (D) TFAP2C knockdown in the T3M4 cell line had a trend of accelerating the G1-S transition of the cell cycle, but this transition rate was not significantly different between the knockdown cells and control cells. The data are presented as the means ± SD (Student’s t-test; *, *P* < 0.05). (PNG 406 kb)

## References

[1] Siegel RL, Miller KD, Jemal A (2017). Cancer statistics, 2017. CA Cancer J Clin.

[2] Von Hoff DD, Ervin T, Arena FP, Chiorean EG, Infante J, Moore M, Seay T, Tjulandin SA, Ma WW, Saleh MN (2013). Increased survival in pancreatic cancer with nab-paclitaxel plus gemcitabine. N Engl J Med.

[3] Conroy T, Desseigne F, Ychou M, Bouche O, Guimbaud R, Becouarn Y, Adenis A, Raoul JL, Gourgou-Bourgade S, de la Fouchardiere C (2011). FOLFIRINOX versus gemcitabine for metastatic pancreatic cancer. N Engl J Med.

[4] Xiong G, Feng M, Yang G, Zheng S, Song X, Cao Z, You L, Zheng L, Hu Y, Zhang T (2017). The underlying mechanisms of non-coding RNAs in the chemoresistance of pancreatic cancer. Cancer Lett.

[5] Bloomston M, Frankel WL, Petrocca F, Volinia S, Alder H, Hagan JP, Liu CG, Bhatt D, Taccioli C, Croce CM (2007). MicroRNA expression patterns to differentiate pancreatic adenocarcinoma from normal pancreas and chronic pancreatitis. JAMA.

[6] Ohuchida K, Mizumoto K, Lin C, Yamaguchi H, Ohtsuka T, Sato N, Toma H, Nakamura M, Nagai E, Hashizume M (2012). MicroRNA-10a is overexpressed in human pancreatic cancer and involved in its invasiveness partially via suppression of the HOXA1 gene. Ann Surg Oncol.

[7] Wang Y, Liu Z, Yao B, Dou C, Xu M, Xue Y, Ding L, Jia Y, Zhang H, Li Q (2016). Long non-coding RNA TUSC7 acts a molecular sponge for miR-10a and suppresses EMT in hepatocellular carcinoma. Tumour Biol.

[8] Yu T, Liu L, Li J, Yan M, Lin H, Liu Y, Chu D, Tu H, Gu A, Yao M (2015). MiRNA-10a is upregulated in NSCLC and may promote cancer by targeting PTEN. Oncotarget.

[9] Chang CH, Fan TC, Yu JC, Liao GS, Lin YC, Shih AC, Li WH, Yu AL (2014). The prognostic significance of RUNX2 and miR-10a/10b and their inter-relationship in breast cancer. J Transl Med.

[10] Long MJ, Wu FX, Li P, Liu M, Li X, Tang H (2012). MicroRNA-10a targets CHL1 and promotes cell growth, migration and invasion in human cervical cancer cells. Cancer Lett.

[11] Jia H, Zhang Z, Zou D, Wang B, Yan Y, Luo M, Dong L, Yin H, Gong B, Li Z (2014). MicroRNA-10a is down-regulated by DNA methylation and functions as a tumor suppressor in gastric cancer cells. PLoS One.

[12] Khan S, Wall D, Curran C, Newell J, Kerin MJ, Dwyer RM (2015). MicroRNA-10a is reduced in breast cancer and regulated in part through retinoic acid. BMC Cancer.

[13] Sun W, Ma Y, Chen P, Wang D (2015). MicroRNA-10a silencing reverses cisplatin resistance in the A549/cisplatin human lung cancer cell line via the transforming growth factor-beta/Smad2/STAT3/STAT5 pathway. Mol Med Rep.

[14] Hoppe R, Achinger-Kawecka J, Winter S, Fritz P, Lo WY, Schroth W, Brauch H (2013). Increased expression of miR-126 and miR-10a predict prolonged relapse-free time of primary oestrogen receptor-positive breast cancer following tamoxifen treatment. Eur J Cancer.

[15] Hudson J, Duncavage E, Tamburrino A, Salerno P, Xi L, Raffeld M, Moley J, Chernock RD (2013). Overexpression of miR-10a and miR-375 and downregulation of YAP1 in medullary thyroid carcinoma. Exp Mol Pathol.

[16] Chen W, Tang Z, Sun Y, Zhang Y, Wang X, Shen Z, Liu F, Qin X (2012). miRNA expression profile in primary gastric cancers and paired lymph node metastases indicates that miR-10a plays a role in metastasis from primary gastric cancer to lymph nodes. Exp Ther Med.

[17] Inoue N, Isomoto H, Matsushima K, Hayashi T, Kunizaki M, Hidaka S, Machida H, Mitsutake N, Nanashima A, Takeshima F (2010). Down-regulation of microRNA 10a expression in esophageal squamous cell carcinoma cells. Oncol Lett.

[18] Li X, Zhang Y, Zhang Y, Ding J, Wu K, Fan D (2010). Survival prediction of gastric cancer by a seven-microRNA signature. Gut.

[19] Cyr AR, Kulak MV, Park JM, Bogachek MV, Spanheimer PM, Woodfield GW, White-Baer LS, O'Malley YQ, Sugg SL, Olivier AK (2015). TFAP2C governs the luminal epithelial phenotype in mammary development and carcinogenesis. Oncogene.

[20] Spanheimer PM, Woodfield GW, Cyr AR, Kulak MV, White-Baer LS, Bair TB, Weigel RJ (2013). Expression of the RET proto-oncogene is regulated by TFAP2C in breast cancer independent of the estrogen receptor. Ann Surg Oncol.

[21] Perkins SM, Bales C, Vladislav T, Althouse S, Miller KD, Sandusky G, Badve S, Nakshatri H (2015). TFAP2C expression in breast cancer: correlation with overall survival beyond 10 years of initial diagnosis. Breast Cancer Res Treat.

[22] Gee JM, Eloranta JJ, Ibbitt JC, Robertson JF, Ellis IO, Williams T, Nicholson RI, Hurst HC (2009). Overexpression of TFAP2C in invasive breast cancer correlates with a poorer response to anti-hormone therapy and reduced patient survival. J Pathol.

[23] Kang J, Kim W, Lee S, Kwon D, Chun J, Son B, Kim E, Lee JM, Youn H, Youn B (2017). TFAP2C promotes lung tumorigenesis and aggressiveness through miR-183- and miR-33a-mediated cell cycle regulation. Oncogene.

[24] Chang TH, Tsai MF, Gow CH, Wu SG, Liu YN, Chang YL, Yu SL, Tsai HC, Lin SW, Chen YW (2017). Upregulation of microRNA-137 expression by slug promotes tumor invasion and metastasis of non-small cell lung cancer cells through suppression of TFAP2C. Cancer Lett.

