# Correction to: MiR-10a-5p targets TFAP2C to promote gemcitabine resistance in pancreatic ductal adenocarcinoma

**DOI:** 10.1186/s13046-021-02152-w

**Published:** 2021-11-18

**Authors:** Guangbing Xiong, Hua Huang, Mengyu Feng, Gang Yang, Suli Zheng, Lei You, Lianfang Zheng, Ya Hu, Taiping Zhang, Yupei Zhao

**Affiliations:** 1 Department of General Surgery, Peking Union Medical College Hospital, Chinese Academy of Medical Sciences and Peking Union Medical College, No. 1 Shuaifuyuan, Wangfujing Street, Beijing, 100730 China; 2 Department of Biliary-Pancreatic Surgery, Affiliated Tongji Hospital, Tongji Medical College, Huazhong University of Science and Technology, Wuhan, 430030 Hubei Province China; 3 Department of Nuclear Medicine, Peking Union Medical College Hospital, Chinese Academy of Medical Sciences and Peking Union Medical College, Beijing, 100730 China; 4 Clinical Immunology Center, Chinese Academy of Medical Sciences and Peking Union Medical College, No. 1 Shuaifuyuan, Wangfujing Street, Beijing, 100730 China

**Correction to: J Exp Clin Cancer Res 37, 76 (2018)**

**https://doi.org/10.1186/s13046-018-0739-x**

Following publication of the original article [1], the authors identified minor errors in Fig. 2; specifically, in Fig. 2b, in the fourth group, the invasion, SU86.86, inhibitor images have been replaced with the correct images.

The corrected figure is provided here. The correction does not have any effect on the results or conclusions of the paper. The original article has been corrected.

**Fig. 2:**
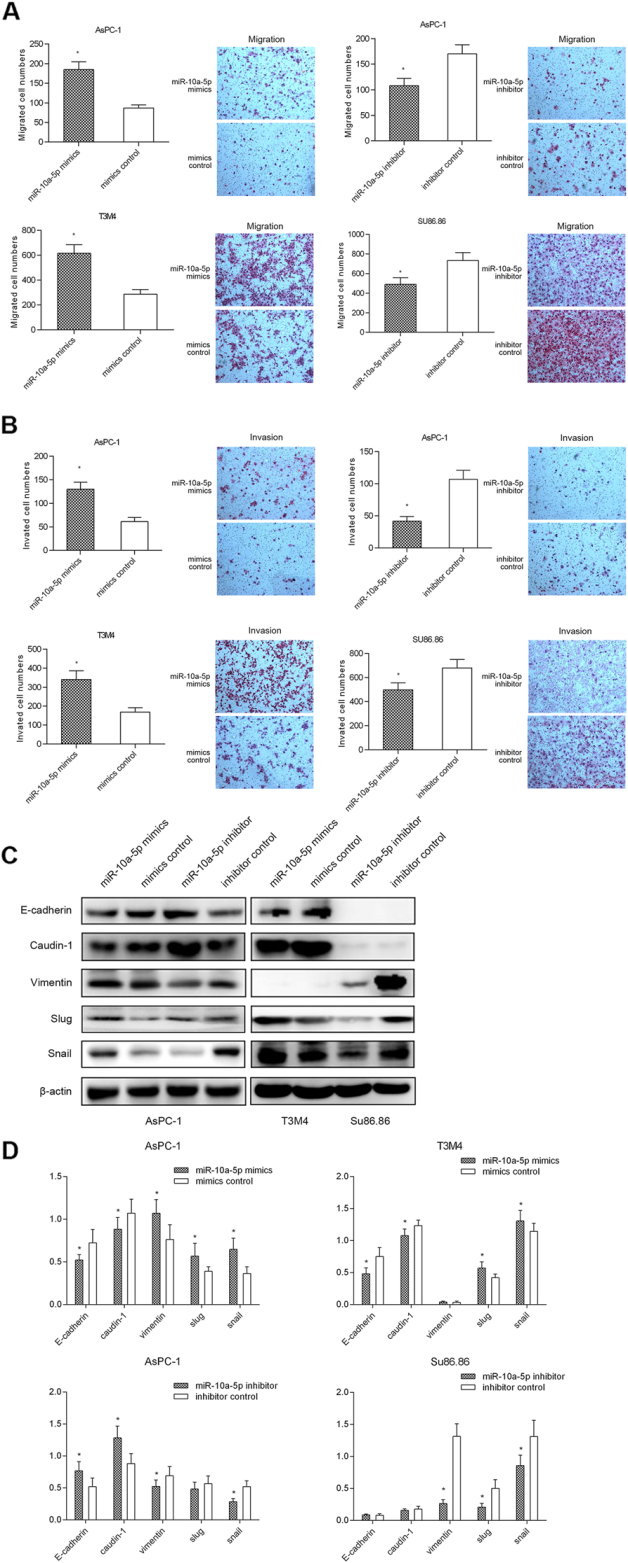
MiR-10a-5p promotes PDAC cell migration and invasion. **a** MiR-10a-5p overexpression promoted T3M4 and AsPC-1 cell migration, while miR-10a-5p knockdown decreased Su86.86 and AsPC-1 cell migration; **b** miR-10a-5p overexpression promoted T3M4 and AsPC-1 cell invasion, while miR-10a-5p knockdown decreased Su86.86 and AsPC-1 cell invasion; **c** miR-10a-5p expression up-regulation decreased E-cadherin and Caudin-1 protein levels while increasing Vimentin, Slug and Snail levels, as determined by using western blotting. **d** The relative intensity of the grayscale band values revealed the changes with miR-10a-5p overexpression or knockdown in PDAC cells; the data are presented as the means ± SD (Student’s t-test; *, *P* < 0.05)
