# Supplementary figures and images for: MiR-10a-5p targets TFAP2C to promote gemcitabine resistance in pancreatic ductal adenocarcinoma
Source: J Exp Clin Cancer Res. 2018 Apr 3;37:76. doi: 10.1186/s13046-018-0739-x (PMC5883523; doi:10.1186/s13046-018-0739-x)

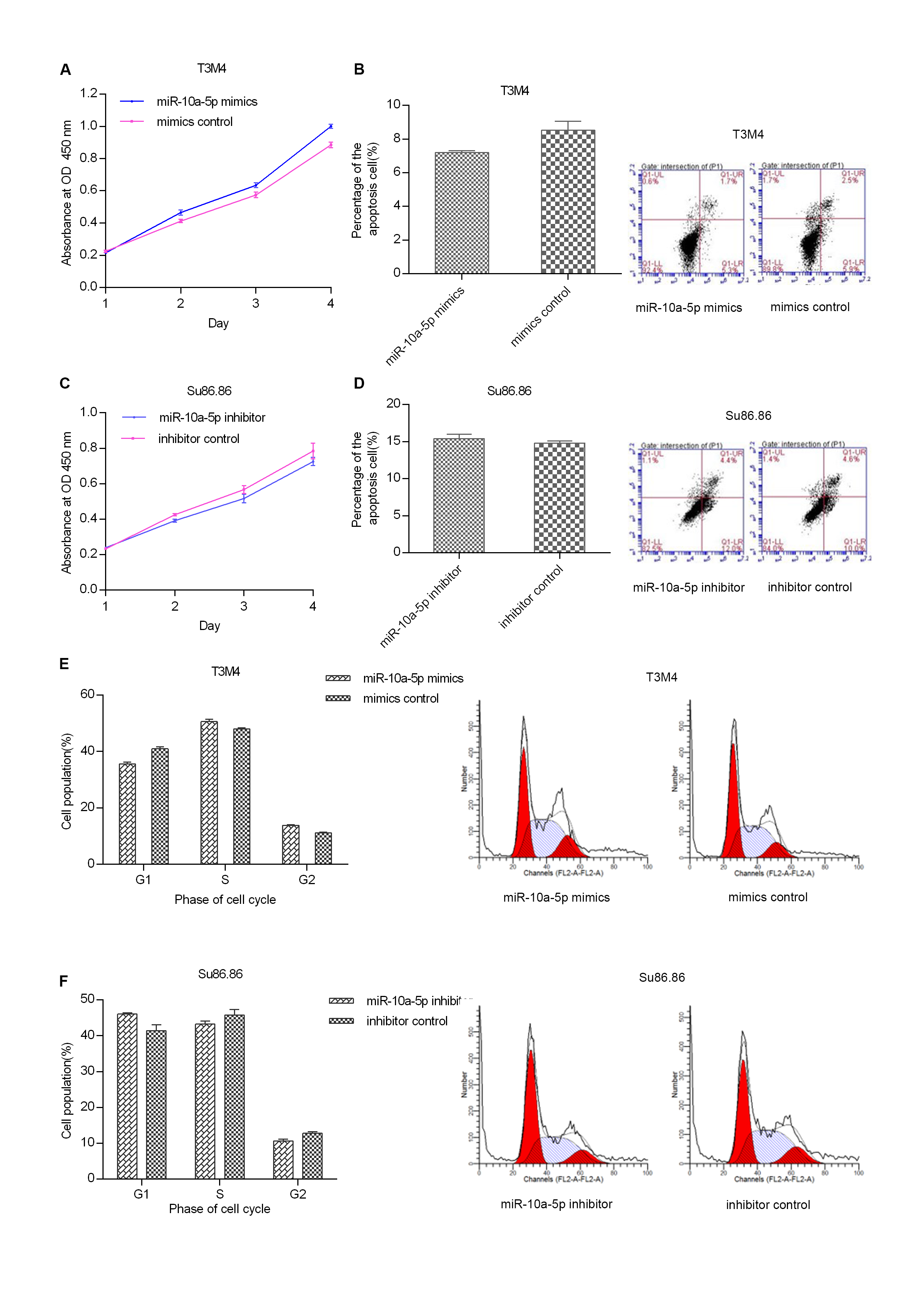

Supplement: Supplementary file 1 — Figure S1. MiR-10a-5p does not influence cell proliferation, cell apoptosis or the cell cycle. (A) MiR-10a-5p overexpression in the T3M4 cell line had a trend of promoting cell proliferation, but proliferation was not significantly different between the overexpressing cells and control cells. (B) MiR-10a-5p overexpression in the T3M4 cell line had a trend of decreasing gemcitabine-induced cell apoptosis, but apoptosis was not significantly different between the overexpressing cells and control cells. (C) MiR-10a-5p knockdown in the Su86.86 cell line had a trend of suppressing cell proliferation, but proliferation was not significantly different between the knockdown cells and control cells. (D) MiR-10a-5p knockdown in the Su86.86 cell line had a trend of promoting gemcitabine-induced cell apoptosis, but apoptosis was not significantly different between the knockdown cells and control cells. (E) MiR-10a-5p overexpression in the T3M4 cell line had a trend of accelerating the S-G2 transition of the cell cycle, but this transition rate was not significantly different between the overexpressing cells and control cells. (F) MiR-10a-5p knockdown in the Su86.86 cell line reduced the S-G2 transition of the cell cycle. The data are presented as the means ± SD (Student’s t-test; *, P < 0.05). (PNG 994 kb) [file 13046_2018_739_MOESM1_ESM.png]

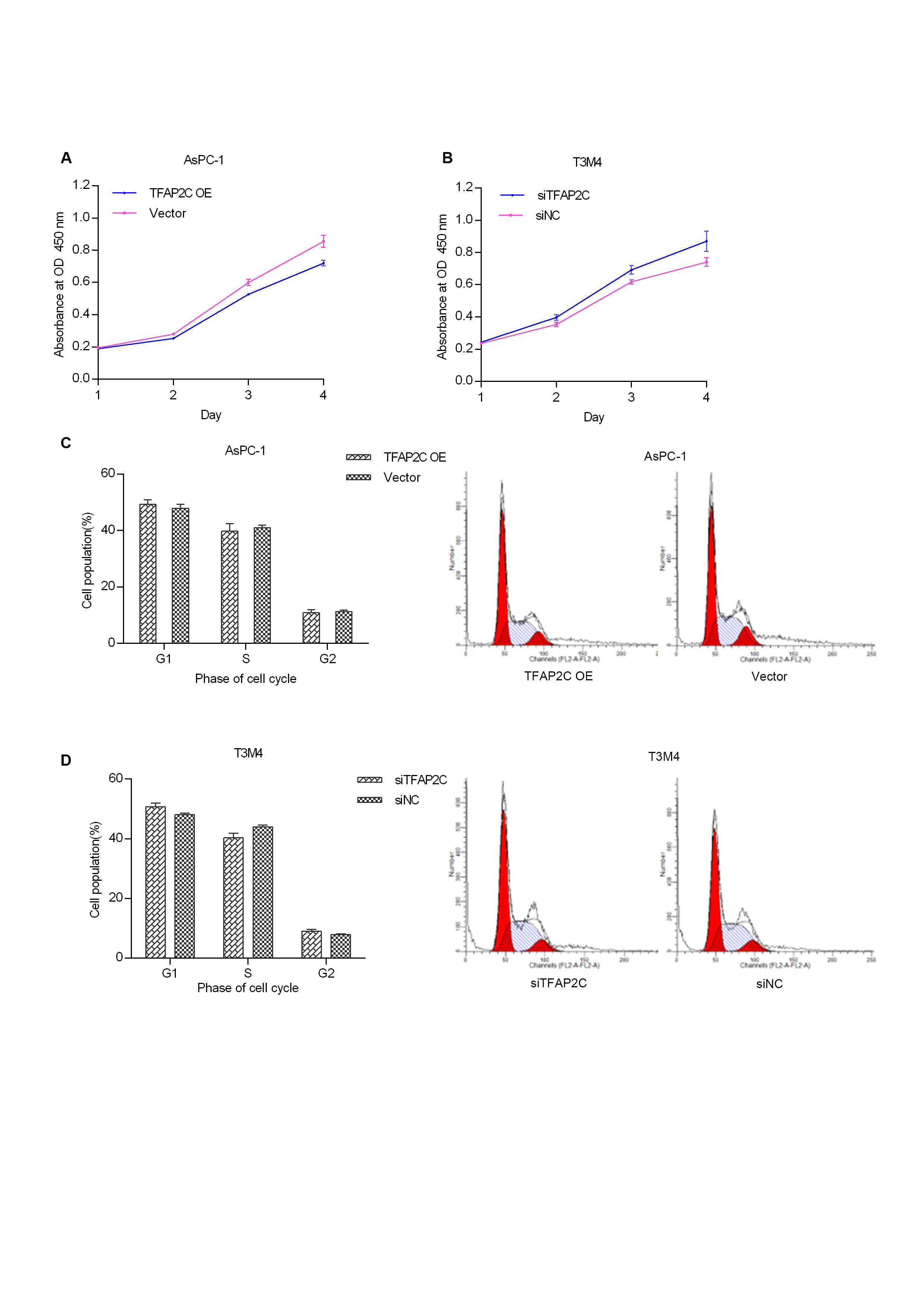

Supplement: Supplementary file 2 — Figure S2. TFAP2C does not influence cell proliferation or the cell cycle. (A) TFAP2C overexpression in the AsPC-1 cell line had a trend of suppressing cell proliferation, but proliferation was not significantly different between the overexpressing cells and control cells. (B) TFAP2C knockdown in the T3M4 cell line had a trend of promoting cell proliferation, but proliferation was not significantly different between the knockdown cells and control cells. (C) TFAP2C overexpression in the AsPC-1 cell line had a trend of reducing the G1-S transition of the cell cycle, but this transition rate was not significantly different between the overexpressing cells and control cells. (D) TFAP2C knockdown in the T3M4 cell line had a trend of accelerating the G1-S transition of the cell cycle, but this transition rate was not significantly different between the knockdown cells and control cells. The data are presented as the means ± SD (Student’s t-test; *, P < 0.05). (PNG 406 kb) [file 13046_2018_739_MOESM2_ESM.png]
